# Racial Disparities in Survival and Age-Related Outcome in Postsurgery Breast Cancer Patients in a New York City Community Hospital

**DOI:** 10.1155/2014/694591

**Published:** 2014-02-12

**Authors:** Stacey Martindale, Awinder Singh, Hua Wang, Ashley Steinberg, Amer Homsi, Haidi Zhang, Alan Go, Peter Pappas

**Affiliations:** ^1^Department of Surgery, The Brooklyn Hospital Center, 121 Dekalb Avenue, Brooklyn, NY 11201, USA; ^2^Department of Surgery, Thomas Jefferson University Hospital, Center City Campus, 111 South 11th Street, PA 19107, USA; ^3^Department of Surgery, Westchester Medical Center, New York Medical College, 100 Woods Road, Valhalla, NY 10595, USA

## Abstract

Breast cancer survival has significantly improved over the past two decades. However, the diagnosis of breast cancer is lower and the mortality rate remains higher, in African American women (AA) compared to Caucasian-American women. The purpose of this investigation is to analyze postoperative events that may affect breast cancer survival. This is a retrospective analysis of prospectively collected data from The Brooklyn Hospital Center cancer registry from 1997 to 2010. Of the 1538 patients in the registry, 1226 are AA and 269 are Caucasian. The study was divided into two time periods, 1997–2004 (period A) and 2005–2010 (period B), in order to assess the effect of treatment outcomes on survival. During period A, 5-year survival probabilities of 75.37%, 74.53%, and 78.70% were seen among all patients, AA women and Caucasian women, respectively. These probabilities increased to 87.62%, 87.15% and 89.99% in period B. Improved survival in AA women may be attributed to the use of adjuvant chemotherapy, radiation, and hormonal therapy. Improved survival in Caucasian patients was attributed to the use of radiation therapy, as well as earlier detection resulting in more favorable tumor grades and pathological stages.

## 1. Introduction

In the USA, breast cancer is the most commonly diagnosed malignancy in women. In 2010, it is estimated that approximately 200,000 women were newly diagnosed with breast cancer and, currently, 1 in 8 women will be diagnosed within their lifetimes [1].

The mortality picture and survival rate associated with breast cancer have been improving over the past two decades. These improvements have been reported to be related to early detection, adjuvant therapy, and radiation therapy [2–4]. However, despite overall improvement in survival, several reports indicate that African American (AA) females have poorer outcomes compared to Caucasian females [5].

Data from the National Cancer Institute's Surveillance, Epidemiology, and End Results (SEER) program indicate that age-adjusted breast cancer incidence rates in African Americans are substantially lower than those from Caucasian women with 141 cases per 100 000 in Caucasian women and 122 in African Americans [6, 7].

Although the incidence may be lower in AAs, the mortality rate appears to be higher compared to Caucasian women [8–10]. Numerous studies have proposed several theories to account for the racial differences in survival. Epidemiologically, AA women are diagnosed at a more advanced stage [11], have tumor-related characteristics that are more commonly estrogen receptor negative, and present with higher grade tumors compared to Caucasian women [12–15]. Socioeconomically, access to health insurance, medical care, and variability in the aggressiveness of treatment have all been proposed as possible contributing factors [16, 17]. Finally, several investigations report that more AA breast cancer patients die from their medical comorbidities than from complications of their breast cancer [18].

A large majority of AA females receive their medical care from community hospitals. Few reports on racial disparities have investigated the role of community hospital care delivery and their effect on outcomes. We conducted a retrospective analysis of prospectively collected data from The Brooklyn Hospital Center's (TBHC) cancer registry. TBHC is an inner city, community-based teaching hospital with a high percentage of AA patients. The study was divided into two time periods, 1997–2004 (period A) and 2005–2010 (period B). We limited our data analysis to postsurgery patients in an attempt to minimize the differences in inequality of access to medical facilities between different racial groups.

Our primary aim was to assess whether known and widely used clinical breast cancer biological factors would explain differences in breast cancer postsurgical survival over the two time periods. Our objective was to determine if racial disparities existed and whether or not these disparities continue to exist. In addition, we sought to identify any factors associated with racial disparities that could serve as points of possible interventions at the healthcare system level.

## 2. Material and Method

### 2.1. Study Population

A retrospective study was conducted by Department of Surgery at TBHC. Data was prospectively collected from TBHC cancer registry from 1997 to 2010. Of the 1537 registered patients, 1276 (79.5%) are African Americans, 281 (17.5%) are white, and 42 (2.9%) are Asian. 1337 subjects underwent surgery.

Demographic data collected include age at diagnosis, sex, race (self-reported), and insurance status. Clinical data such as stage at diagnosis, tumor location, mode of diagnosis, histopathology, pathology stages, lymph nodes status, ER, and PR status (available after 2003), and methods of treatment were also included. Those who did not undergo surgery were not included in the survival analysis. The study was approved by the local Institutional Review Board at TBHC.

### 2.2. Study Design

The study was divided into two time periods, 1997–2004 (period A) and 2005–2010 (period B). Baseline characteristics were compared between the two different periods and between the two racial groups. These comparisons include distribution of the histological grade, pathological stages, surgery, chemotherapy, ER, and PR status. Age was subdivided into three subgroups <45, 45–60, and >60 for survival analysis. Survival was compared between these two time periods in AA and Caucasian women, respectively. Based on the baseline characteristics and survival improvement between the two time periods, we compared whether there are survival disparities between the two populations.

### 2.3. Outcome Measures

The follow-up cutoff was on October 31, 2010. Overall survival was calculated from the date of diagnosis to the data of death from any causes or the follow-up cutoff.

### 2.4. Statistical Analysis

Microsoft Excel and Access were used for database management. Statistical analyses were done with SPSS (SPSS Inc., Chicago, IL, USA) version 16 and Epi info 3.5.3 (Center for Disease Control and Prevention). Chi square and Fisher's exact test were used to examine the distribution of cancer location, histological grade pathological stages, surgery versus nonsurgery, and chemotherapy versus nonchemotherapy. One-way analysis of variance (ANOVA) was conducted to compare the difference in age at diagnosis between AA and Caucasian women. Kaplan-Meier survival probabilities and multivariate Cox proportional hazards models were applied to estimate hazard ratios (HR) with 95% confidence intervals (95% CI).

## 3. Results

### 3.1. Baseline Characteristics

The average age at diagnosis is 61.04 (SD ± 13.99) in period A and 59.95 (SD ± 13.80) in period B (*P* > 0.05). Caucasian females were diagnosed at a younger age than AA in period B suggesting earlier detection; however, no significant differences were observed (see Table 1).

Significant differences in histological grade as well as pathology stages between the two time periods were observed. As there are approximately 21% of cell type reported as undetermined in histological grade in our registry and 10-11% unstaged in pathology, our data analysis was performed twice. The data presented here excluded cell type undetermined and upstaged. We observed a high percentage of grades I and II carcinomas in period A and a high percentage of grades III and IV carcinomas in period B in the overall total population. When we analyzed the population according to race, a high percentage of high-grade differentiation was present in period B in AA compared to Caucasian women. White females demonstrated more grade II cancers in period B and more grade III cancers in period A, suggesting earlier detection in Caucasian females in the latter time period compared to AAs (see Table 1). We repeated our data analysis and included the cell type undetermined, which still demonstrated a high percentage of high-grade cancer in period B in AAs.

For the pathology stages, no significant differences between the two time periods were observed in AAs and Caucasian females combined together and AAs alone. Significant differences in breast cancer staging on presentation were observed in the Caucasian group during the two time periods. A higher percentage of stage 0 and stage I cancers was observed in Caucasian females in period B versus a high percentage of stage II and stage III cancers in period A (*P* < 0.003).

Significant difference was observed in the ER/PR status with a high percentage of ER positive in period B: 37/200 (19.0%) compared with 46/401 (11%) positive in period A (*P* = 0.024) as well as high percentage of PR positive in the period B, 50/255 (20%) compared with 34/145 (11) in period A (*P* < 0.001) (see Table 1) in the overall cohort. No significant differences in Caucasian females were observed in ER (*P* = 0.405) and PR (*P* = 0.476) status in the two time periods. For AA females, a significant difference in both ER (*P* = 0.025) and PR (*P* < 0.001) was observed (see Table 1).

Among all the operative procedures performed, the two most common were modified radical mastectomy (MRM) and lumpectomy. For the MRM, there were 33.1% (early period) versus 31.4% (late period) and for lumpectomy there were 27.9% (early period) versus 39.1% (late period). 21.6% versus 0.8% of patients had simple mastectomies during periods A and B, respectively.

The percentage of patients receiving chemotherapy, radiation therapy, and hormone therapy increased in period B (see Table 2). Significant differences were observed in AA in all three therapies with *P* < 0.001, *P* = 0.007, and *P* = 0.018, respectively. Marginal differences were observed in Caucasian females for radiation therapy and trend in chemotherapy.

### 3.2. Postsurgery Survival Improvement: 1997–2004 versus 2005–2010

Kaplan Meier survival analysis: postsurgery survival probability significantly improved when comparing 1997–2004 versus 2005–2010 periods (Log-rank *P* < 0.001). Survival probability increased 12.25% in period B (AA + White); the increase in survival is greater in AA females than in Caucasian females with an increase of 12.62% in AA compared with 11.29% in White. Overall 5-year survival probabilities, survival among AA women and survival among Caucasian women are compared in Table 3.

During period A, 5-year survival probabilities of 75.37%, 74.53%, and 78.70% were seen among all patients, AA women and Caucasian women, respectively. These probabilities increased to 87.62%, 87.15%, and 89.99% in period B. Cox regression hazard ratio is 2.1824. This postsurgery survival improvement was not affected by grade, site of tumor, and chemotherapy, but it was associated with pathology stages and number of lymph nodes removed.

### 3.3. Racial Disparity in Outcome

An increase in survival was observed between periods A and B. Kaplan Meier survival analysis did not demonstrate a difference in overall survival between AA (78.94%) and Caucasians (82.42%) (*P* = 0.5187). However, when patient age was divided into three subgroups, a significant difference was observed in survival in patients aged 45–60 years (Log-rank *P* = 0.05), with 5 years of survival probability in African American being 86.98% and in White being 92%. This disparity is more obvious in early periods with log-rank *P* = 0.047 but not present in late periods (data not shown here). Cox hazard ratio 1.90, 97.61% confidence, interval 3.72, pathological stages, and the number of lymph nodes removed were associated with the prognosis (*P* = 0.052). However, there is no difference of the distribution in terms of the tumor grade, pathological stages, and ER/PR status between African American and White in this age group (data not shown here).

## 4. Discussion

In this retrospective, single-institution study, we found a significant improvement in survival between the two time periods in both populations. However, the baseline characteristics and treatment options that potentially contributed to the improvement are apparently different between these two ethnic groups. Age-specific racial disparity in outcomes was observed.

### 4.1. Baseline Characteristics Difference between AA and White

There is no significant difference for the age of patients at diagnosis between the two time periods. An interesting baseline characteristic difference in our study is the histological grade. Histological grade has been considered as an independent risk factor for the prediction of survival. It has been reported that AAs have a higher percentage of poor grade tumors compared to Caucasian females. Considering the very significant survival improvement over the two time periods, we expected that the tumor would be detected in earlier TNM stages with a low-grade presentation. We have observed the opposite direction.

As undetermined tumor grades were more prevalent in period A, we cannot exclude the possibility that the low number tumors with this characteristic may represent a sample bias rather than a change in tumor biology. However, our study also demonstrated that, in the early TNM stages with negative lymph nodes, histology grade was not an independent predictor for survival [19]. So it is not a certainty that low grade is necessarily associated with a good outcome. The most recent study based on the molecular biomarker level reported that several molecules such as TOP2A, MCM2, and BUB1B proteins are potential molecular biomarkers of malignancy in histologically normal breast tissue and benign breast tissues may indicate a poor prognosis [20]. Characterizing tumors at the molecular level may be more accurate in predicting outcomes.

We have also observed that significant differences were observed only in Caucasian females with a high percentage of early stages in period B and advanced stages in period A. TNM pathology stages have been considered the most precise predictive factor for prognosis. It is not difficult to understand that with increased public awareness more and more tumors are detected in the early stages, which leads to early treatment and significant improvement in survival [21, 22]. However, there is no significant difference in pathology stages in AA between the two time periods. A possible reason for this observation is that there is no significant change in terms of the screening rate or follow-up after screening in AA population. Adams reported that AA women were 12% less likely than European American women to complete the recommended workup after mammography [23]. This may explain why there was no significant difference observed in the pathological stages between the two time periods for AA women, suggesting that survival improvement in the AA population may be attributed to other changes.

ER+ and PR+ are important prognostic risk factors for survival. Increased percentage of ER and PR positive tumors was observed in period B in both AAs and Caucasian females. ER and PR positive status indicates better prognosis which can account for the survival improvement in period B. However, the correlation between ER and PR status with histological grade is not clear. In our study, we observed that histological grade in period B is higher compared with period A in AA population which contrasts to the ER and PR status. Several studies try to identify the possible association between ER and PR status with histological grade. Correlation between ER and PR positivity and histological grade has been reported in previous investigations [24, 25]. ER positive tumors were more likely than ER negative tumors to demonstrate histological evidence of tumor differentiation [26], and the better-differentiated tumors rarely lacked the receptor, although this correlation was significant only in women defined as postmenopausal [27]. However, at the molecular level, studies report that ER negative breast cancers have unique characteristics: ER-negative tumors show a higher expression of p53, CerbB2, and epidermal growth factor receptor compared to ER-positive breast cancer [28] and these unique features support the concept that ER-negative tumors are a morphologically and phenotypically distinct entity, which are independent of histological grade. Several molecules such as TOP2A, MCM2, and BUB1B proteins are reported as potential molecular biomarkers of malignancy presented in histological normal and benign breast tissues, which indicate poor prognosis [21]. Based on these studies, current new concept considered that breast cancer is a heterogeneous disease characterized by varied morphological appearances, molecular features, behavior, and difference response to therapy [29].

### 4.2. Survival Improvement Difference between AA and White

Significant improvements have been achieved in both groups over the two periods. Based on the baseline characteristics, the factors that attributed to the improvement are different between these two groups. Although surgery is considered the primary treatment for breast cancer and many patients with early stage breast cancer are cured with surgery alone, early detection through mammography, adjuvant therapy, and radiation therapy are considered the three most important factors that have contributed to overall survival improvements for the past two decades. Studies indicate that adjuvant treatments have improved survival in early stages of breast cancer [30], metastatic breast cancer [31], and recurrent breast cancer [3]. Radiation therapy decreases local recurrence and improves survival as well [4, 32–34].

While there were no significant differences in pathologic stages for AA women between the two periods, significant differences were observed as far as the increased use of chemotherapy, adjuvant and radiation therapy in this population. Based on that, our data suggests that improved survival in AAs is likely related to the use of these therapies, as opposed to early detection. As significant difference for pathology stages was observed in Caucasian females, survival improvement appears to be related to early detection in addition to adjuvant therapies. Our results are supported by several reports. Burton et al. reported that survival improvements were not related to mammographic screening efforts and concluded that adjuvant therapies must be responsible for survival benefits [35]. Adjuvant hormonal and chemotherapeutic administration has increased in Australia since 1986, whereas mammographic rates have remained unchanged. A report [36] studying 5 neighboring European countries with different levels of screening but similar access to treatment revealed that the contrast between the time differences in implementation of mammography screening and the similarity in reductions in mortality between the country pairs suggest that screening did not play a direct part in the reductions in breast cancer mortality.

### 4.3. Racial Disparity in Outcome between AA and White

As an exploratory analysis to compare two ethnic groups over two time periods in survival in postoperative patients, we also investigated whether racial disparity affects outcomes. Studies show that racial disparity in survival did exist between AA and Caucasian females. Although several authors state that biological factors, access to medical facility, and social economic status contribute to racial disparity [37–39], other investigations report that racial disparity does not play a major role in outcomes. Our data also shows an age-specific disparity in the 40-to 59-year-old females, despite to absence of significant difference of the baseline characteristics of AA and Caucasian females in this age ground, suggesting that there exists other factors that contribute to this disparity.

A study from Natarajan [40] and colleagues analyze breast cancer survival by race, 2,296 black and 24,265 white from 565 hospitals in a long-term survival. Race remained a prognostic factor after adjusting for stage, age, and tumor characteristics, which mean survival differences are only partially explained by differences in stages and related factors.

Newman et al. [41] identified 20 studies from 1980 to 2005. After adjusting for age, stage, and socioeconomic status, they reported that African American ethnicity was associated with a statistically significant increased mortality risk in overall survival (mortality hazard, 1.27; 95% CI, 1.18 to 1.38) and in breast-cancer-specific survival (mortality hazard, 1.19; 95% CI, 1.10 to 1.29). Whitman et al. [9] calculated age-adjusted breast cancer mortality rates for women in Chicago, New York City, and the USA from 1980 to 2005. The rate ratios were approximately equal in 1980 and stayed that way until the early 1990s, when the rates started to decline while the AA rates remained rather constant.

Our study demonstrated age-related racial disparity and the differences were most pronounced in period A, which is in agreement with Deshpande et al. [42]. These investigators conducted a retrospective, population-based cohort study SEER data from 1988 to 2003. They compared overall and stage-specific breast-cancer mortality between Black and White women within each age (<40, 40–49, 50–64, and 65+) and stage (stage 0-IV and unstaged) group at diagnosis. Racial disparities in breast-cancer-specific mortality were predominantly observed within each stage at diagnosis among women < 65 years old.

As we could not find any biological difference between these two groups to explain what we have observed, some other possibilities need to be considered. One limitation of our study is the lack of enough information for the comorbidities such as obesity which may has the potential to impact racial disparity outcomes. One phenomenon that cannot be ignored is the rapid increase of the obesity in the USA. The National Healthcare Quality Report from US Department of Health and Human Services Office of Minority Health states that African American women have the highest rates of being overweight or obese compared to other groups in the USA [43]. Of them, middle-aged adults are more likely to be obese than Americans of other ages or ethnic groups [44]. More investigations are reporting that obesity is an independent risk factor for the development of breast cancer, and it has been associated with a poor outcome [45, 46]. Furthermore, obesity is an independent prognostic factor for developing distant metastases and the effects of adjuvant therapy seem to be lost more rapidly in patients with obesity [47]. Most studies report that obesity is correlated with breast cancer in postmenopausal women; however, study from population-based sample of 1,360 Australian women with breast cancer indicated that obesity is independently associated with poorer outcomes in premenopausal women, as it is in postmenopausal women [48].

Therefore, we could not exclude the possibility that obesity and/or other comorbidities such as diabetes may attribute to this racial disparity in outcome in our study. Several studies have observed adipocytes in close proximity to invasive cancer cells and have been referred to as cancer-associated adipocytes (CAAs). These cells are considered to be essential for breast tumor development/progression through molecular crosstalk with their invasive cancer cell counterparts [49]. Obesity-related effects on insulin levels and the insulin-like growth factor-1 (IGF-1) axis, some adipokines, and inflammatory cytokines also stimulate breast cancer growth and metastasis, both directly and most probably by enhanced angiogenesis [50]. Excess adipose tissue in obese women leads to increased production of estrogen. Exposure to estrogen is an important determinant of the risk of breast cancer, as it stimulates the growth of tissue and its metabolites have been found to be genotoxic and mutagenic [51]. An interesting study shows that body fat distribution may be a better marker of a hormonal pattern associated with increased breast cancer risk than obesity; obese premenopausal African American (AA) women with upper body fat (UBF) phenotype have a high-risk hormonal profile [52, 53]. Moreover, study also found that higher BMI was associated with worse pathologic complete response (pCR).

Another limitation of our study is lack of information at molecular level. More and more studies are trying to identify predictive factors at the molecular level which is very promising. Genomic analyses have subclassified breast cancer into 4 categories: luminal A (ER+ and/or PR+, HER2-); luminal B (ER+ and/or PR+, HER2+); HER2+ (ER-, PR-); and basal-like (ER-, PR-, HER2-) [54]. Established molecular biomarkers such as estrogen receptor and progesterone receptor have played a significant role in the hormone treatment. Premenopausal AA has a higher likelihood of developing triple negative cancer and has poorer prognoses [55, 56]. Triple negative breast cancers are increased in AA females regardless of age or body mass index [57]. Although our data indicate that the percentage of ER+ and PR+ have increased over the two time periods which may account for the better prognosis in period B, we did not have data on HER2 status and therefore cannot comment on whether or not HER2 status affected our survival data.

An interesting new theory proposed by Demicheli et al. [58] in his review in which he summarized and described that breast cancer may be a mixture of at least 2 main diseases and/or cause pathways-“2-diseases assumption” [59, 60]. The first one is early onset with peak incidence near age 50 years, overrepresented among AA women compared with Caucasia women and generally more aggressive outcome. The second breast cancer is late onset with peak incidence at age of 70 years, more indolent course. This theory may be one of the possible explanations of the racial disparity in outcome in this age group as observed in our study.

### 4.4. Study Limitations

As an exploratory analysis that focuses on subjects who received surgical procedure based on registry data, there are several limitations. First, this is a retrospective study and, therefore, is subject to all the biases inherent to a retrospective design. Second, is the partially available information for ER and PR status that limited sample size and subsequently reduced the power of the study. No HER2 or obesity data was available. We observed a fair amount of undermined histological grade and unstaged pathology in our dataset. Third, when we divided into age subgroup, the sample sized decreased in the Caucasian female group. In general, we cannot exclude the possibility of sample bias particularly in Caucasian group as the sample size is smaller than the AA group. Fourth is the lack of comorbidity information.

## 5. Conclusions

In conclusion, our study suggests that survival rate significantly improved over the two time periods in both AA and Caucasian females. The survival improvement was greater in AAs than Caucasians. In AAs, the improvement is related to adjuvant and radiation treatments, whereas early screening appeared to have a greater affect on Caucasians. Subgroup analysis indicated that AA survival was decreased in women between the ages of 45 and 60 compared to Caucasian females.

### 5.1. Future Intervention

Although registry studies have limitations, they do provide valuable information regarding the multifactorial differences in breast cancer outcomes among African Americans as compared to Caucasian females. AA women should emphasize prompt initiation of aggressive and specific chemo, adjuvant regimens once diagnosed. Early detection of lower-stage tumors will also potentially reduce breast mortality in AA. More future studies identifying differences in gene expressions will help to target and enhance treatment more specifically, such as 21-gene recurrence score [61]. Genetic reclassification of histologic grade which delineates new clinical subtypes of breast cancer should be considered to implant in clinical practice for better prediction [62]. A better understanding of the breast cancer at molecular level may lead to interventions that reduce racial disparities in breast cancer survival.

## Figures and Tables

**Table 1:** 1997–2004 versus 2005–2010 baseline characteristics comparison.

Variables	1997–2004	2005–2010	*P* value
Mean age at diagnosis			
Overall	61.04 (SD ± 13.99)	59.95 (SD ± 13.80)	0.142
AA	61.0 (SD ± 13.68)	60.11 (SD ± 13.69)	0.261
White	61.23 (SD ± 15.20)	59.29 (SD ± 12.92)	0.281
Race			
AA	650.00	575.00	0.798
White	165.00	104.00	0.202
Grade			
I well	89.00	62.00	
II moderate	213.00	210.00	
III poor	177.00	239.00	
IV undifferential	1.00	29.00	<0.001
Grade-AA			
I well	69.00	54.00	
II moderate	169.00	171.00	
III poor	137.00	214.00	
IV undifferential	1.00	21.00	<0.001
Grade-White			
I well	20.00	8.00	
II moderate	44.00	39.00	
III poor	40.00	25.00	
IV undifferential	0.00	8.00	0.003
Pathology stages			
0	72.00	97.00	
I	143.00	155.00	
II	226.00	220.00	
III	95.00	98.00	
IV	7.00	6.00	0.491
Pathology stages-AA			
0	62.00	75.00	
I	113.00	125.00	
II	166.00	190.00	
III	78.00	90.00	
IV	4.00	6.00	0.982
Pathology stages-White			
0	10.00	21.00	
I	30.00	30.00	
II	60.00	30.00	
III	17.00	8.00	
IV	3.00	0.00	0.003*
ER			
ER+	46.00	37.00	
ER−	355.00	163.00	0.024*
ER-AA			
ER+	45.00	322.00	
ER−	38.00	158.00	0.025*
ER-White			
ER+	6.00	69.00	
ER−	3.00	18.00	0.405
PR			
PR+	34.00	50.00	
PR−	311.00	205.00	<0.001*
PR-AA			
PR+	30.00	292.00	
PR−	53.00	188.00	<0.001*
PR-White			
PR+	5.00	59.00	
PR−	4.00	28.00	0.476

**Table 2:** 1997–2004 versus 2005–2010 treatments comparison

Variables	1997–2004	2005–2010	*P* value
Chemotherapy			
Yes	243.00	296.00	
No	596.00	404.00	<0.001*
Chemotherapy-AA			
Yes	185.00	242.00	
No	465.00	333.00	<0.001*
Chemotherapy-White			
Yes	48.00	41.00	
No	117.00	63.00	0.053

Radiation therapy			
Yes	217.00	244.00	
No	474.00	362.00	0.001*
Radiation therapy-AA			
Yes	176.00	206.00	
No	372.00	308.00	0.007*
Radiation therapy-White			
Yes	41.00	54.00	
No	102.00	38.00	0.049*

Hormone therapy			
Yes	93.00	112.00	
No	598.00	494.00	0.015*
Hormone therapy-AA			
Yes	71.00	94.00	
No	477.00	420.00	0.018*
Hormone therapy-White			
Yes	22.00	18.00	
No	121.00	74.00	0.477

**Table 3:** 5-year survival rate comparison between two time periods.

	1997–2004	2005–2010	Increased %
AA + White	75.37%	87.62%	12.25%
AA	74.53%	87.15%	12.62%
White	78.70%	89.99%	11.29%
AA-White difference	4.17%	2.84%	1.33%
